# Cutoff of the reverse shock index multiplied by the Glasgow coma scale for predicting in-hospital mortality in adult patients with trauma: a retrospective cohort study

**DOI:** 10.1186/s12873-024-00978-z

**Published:** 2024-04-08

**Authors:** Jun Seong Park, Sol Ji Choi, Min Joung Kim, So Yeon Choi, Ha Yan Kim, Yoo Seok Park, Sung Phil Chung, Ji Hwan Lee

**Affiliations:** 1 Department of Emergency Medicine, Yonsei University College of Medicine, 50-1 Yonsei-Ro, Seodaemun-Gu, 03722 Seoul, Republic of Korea; 2 Biostatistics Collaboration Unit, Department of Biomedical Systems Informatics, Yonsei University College of Medicine, Seoul, Republic of Korea

**Keywords:** Triage, Reverse shock index multiplied by the Glasgow coma scale, Trauma, Mortality

## Abstract

**Background:**

Early identification of patients at risk of potential death and timely transfer to appropriate healthcare facilities are critical for reducing the number of preventable trauma deaths. This study aimed to establish a cutoff value to predict in-hospital mortality using the reverse shock index multiplied by the Glasgow Coma Scale (rSIG).

**Methods:**

This multicenter retrospective cohort study used data from 23 emergency departments in South Korea between January 2011 and December 2020. The outcome variable was the in-hospital mortality. The relationship between rSIG and in-hospital mortality was plotted using the shape-restricted regression spline method. To set a cutoff for rSIG, we found the point on the curve where mortality started to increase and the point where the slope of the mortality curve changed the most. We also calculated the cutoff value for rSIG using Youden's index.

**Results:**

A total of 318,506 adult patients with trauma were included. The shape-restricted regression spline curve showed that in-hospital mortality began to increase when the rSIG value was less than 18.86, and the slope of the graph increased the most at 12.57. The cutoff of 16.5, calculated using Youden's index, was closest to the target under-triage and over-triage rates, as suggested by the American College of Surgeons, when applied to patients with an rSIG of 20 or less. In addition, in patients with traumatic brain injury, when the rSIG value was over 25, in-hospital mortality tended to increase as the rSIG value increased.

**Conclusions:**

We propose an rSIG cutoff value of 16.5 as a predictor of in-hospital mortality in adult patients with trauma. However, in patients with traumatic brain injury, a high rSIG is also associated with in-hospital mortality. Appropriate cutoffs should be established for this group in the future.

## Background

According to the report from the World Health Organization, over 4.4 million individuals die annually due to trauma [1]. To reduce trauma-related mortality, various advanced diagnostic and treatment methods, such as multidetector computed tomography and angiographic embolization, are actively being introduced for patients with trauma [2, 3]. However, despite these efforts, the trauma-related death in Korea has been on the rise, increasing from 1,210 in 2014 to 1,647 in 2021 [4]. Therefore, efforts are required to address this upward trend.

Preventable Trauma Death Rate (PTDR) is defined as the death rate that could have been prevented if patients with trauma were successfully transferred to appropriate medical facilities [5–7]. In 2019, Jung et al. reported a PTDR of 30.5% in Korea, which was closely associated with the time required for patients to reach their final treatment facility [6]. Therefore, the early identification and timely transfer of patients at risk of early death during the pre-hospital or initial trauma care stages to trauma centers could be pivotal for reducing PTDR.

Various field triage tools are employed to identify suitable hospitals for treating patients with trauma. However, a systematic review and meta-analysis by Gianola et al. in 2021 found that among the 13 field triage tools used for adult patients with trauma, none met the American College of Surgeons' target of less than 5% under-triage and less than 35% over-triage rates [8, 9]. Hence, there is a need for continued efforts to identify an appropriate field triage tool.

In 2018, Kimura et al. developed the reverse shock index multiplied by Glasgow Coma Scale (GCS) (rSIG) as a trauma assessment tool by multiplying GCS with rSI [10]. The rSIG has been since recognized for its utility in predicting the need for massive transfusion and mortality in trauma patients [11–13], suggesting its potential for identifying severe trauma patients who were at risk of potential death. However, to utilize continuous variables such as rSIG as a screening tool for identifying severe trauma patients, establishing a reliable cutoff is essential. Previous studies have proposed cutoff values of 10.20, 14, and 18 for rSIG to predict in-hospital mortality in trauma [11, 13, 14]. This substantial differences among the previously proposed cutoffs necessitates additional research to refine them. Furthermore, the cutoffs proposed by Lee et al. [11] and Wan-Ting et al. [13] were based on data from patients with severe trauma diagnosed using existing trauma severity screening tools such as field triage guidelines, injury severity scores, or abbreviated injury scales. While cutoffs derived from such populations may aid in prioritizing treatment at trauma centers with a high density of severe trauma patients, such values may be limited when used as cutoffs to decide transfers to trauma centers.

Therefore, we aimed to determine the correlation between in-hospital mortality and rSIG in adult patients with trauma and to identify appropriate cutoff values for predicting in-hospital mortality. Once an appropriate cutoff value is established, it is expected to serve as a criterion for selecting patients for transfer to trauma centers from the emergency rescue scene, potentially contributing to the reduction in PTDR.

## Methods

### Study design

This multicenter retrospective cohort study aimed to determine the rSIG cutoff value for predicting in-hospital mortality in adult patients with trauma. The data used in this study were extracted from the Emergency Department-based Injury In-depth Surveillance (EDIIS) registry collected between January 1, 2011 and December 31, 2020. The EDIIS is a national survey conducted by the Korea Disease Control and Prevention Agency to establish community-based injury prevention policies. It involves the collection of demographic information, injury mechanisms, and treatment outcomes of injury patients presenting to 23 emergency departments in Korea. This study was conducted with the approval of the Yonsei University Health System, Severance Hospital, Institutional Review Board (Approval Number: 4–2023-0470). Because of the retrospective nature of this study using previously collected data, obtaining individual consent from the participants was waived. All study methods were performed in accordance with the relevant guidelines and regulations or declaration of Helsinki.

### Inclusion and exclusion criteria

This study included patients aged 18 years and above, those with acute trauma who arrived at the emergency department within 6 h of the injury, and those transferred through emergency medical system. We defined acute trauma patients as those arriving within 6 h of trauma according to the time criteria defined by Kim et al., whose study was conducted in the same country as our study [15]. Patients with non-traumatic injuries (such as intoxication, drowning, hypothermia, chemical exposure) and those with missing values such as treatment results, systolic blood pressure (SBP), heart rate (HR), GCS, and excessive mortality ratio-adjusted Injury Severity Score (EMR-ISS) were excluded. The EMR-ISS, which reflects trauma severity, has been validated as a tool for assessing trauma severity based on patient diagnosis [16]. Patients transferred from the emergency department to other hospitals for whom treatment outcomes could not be verified and those who died before arriving at the hospital were also excluded from the study.

### Study data and variables

Age and sex were collected as basic demographic data, whereas the time elapsed from injury to hospital arrival and mechanism of trauma were gathered as trauma-related information. The vital signs and GCS for measuring rSIG were collected at the initial arrival stage in the emergency department. Additionally, the EMR-ISS based on electronic medical records was collected as an indicator reflecting the severity of the injury. Cases with unreliable values, such as SBP below 30 mmHg or above 300 mg and HR above 200 beats/min, were treated as missing values. The primary outcome was in-hospital mortality.

### Statistical analysis

Nominal variables are reported as frequencies (%), and continuous variables are presented as medians (interquartile range, IQR). Chi-square tests were used for group comparisons of nominal variables, whereas the Mann–Whitney U test was used for continuous variables because of their non-normal distribution.

Binary logistic regression analysis was conducted to identify the factors contributing to in-hospital mortality. During the regression analysis, SBP, HR, and GCS were excluded because they were variables reflected in the rSIG. Multivariable logistic regression analysis was performed for factors showing associations in the univariable analysis with p < 0.05. The results of each analysis are presented as odds ratios (95% confidence intervals, CI).

Two statistical methods were employed to determine the cutoff values for rSIG. The shape-restricted regression spline method was used to determine the relationship between in-hospital mortality and rSIG. This statistical approach involves plotting a nonlinear curve based on the distribution of predicated values derived from a logistic regression analysis and identifying the point at which the slope of the curve undergoes the most significant change [17]. Furthermore, the coordinates of the curve obtained through this process were scrutinized to calculate the rSIG value at which the in-hospital mortality started to increase. Additionally, the rSIG cutoff was determined using Youden's index, a commonly employed metric for calculating cutoff values. Receiver Operating Characteristic (ROC) curves and the area under the curve (AUC) are presented for each analysis.

The participants were stratified into subgroups based on the presence or absence of traumatic brain injury (TBI), and additional analyses were conducted for each subgroup. TBI group was defined as patients diagnosed with the S06 code (intracranial injury) according to the International Classification of Diseases, 10th Revision. This group encompassed not only patients with isolated TBI but also those with additional traumatic injuries alongside TBI.

Impaired consciousness in trauma patients can occur due to causes other than TBI, such as shock or hypoxemia, making it difficult to definitively distinguish TBI status in the pre-hospital phase. If differences in the correlation between in-hospital mortality and rSIG exist among TBI and non-TBI groups, patients will be divided into three subgroups for additional sensitivity analysis: isolated TBI, TBI with other concomitant injuries, and severe trauma without TBI. Severe trauma patients were defined as those with an EMR-ISS score of 25 or higher according to the EMR-ISS classification criteria [16].

## Results

**Fig. 1:**
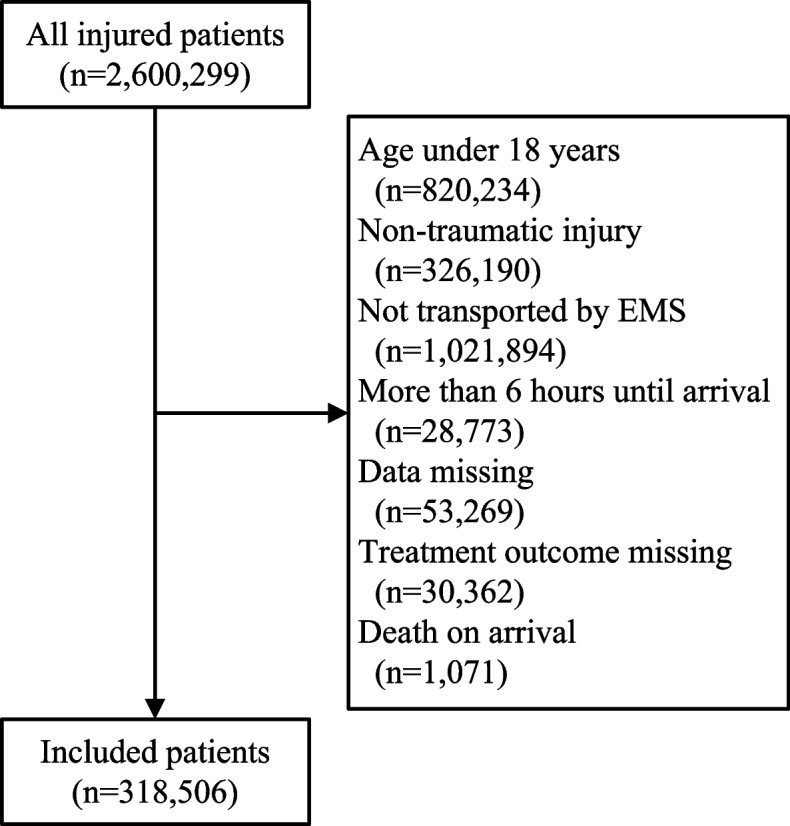
Flow diagram of the inclusion process

During the study period, 2,600,299 patients with trauma visited emergency departments participating in the EDIIS project. Among them, 2,197,091 patients were excluded, including those under 18 years of age and those who did not meet the inclusion criteria, such as those with non-traumatic injuries. Additionally, 84,702 patients with missing essential variables, such as GCS, vital signs, and treatment outcomes, were excluded. Data from 318,506 patients were included in the final analyses. Among these, the number of in-hospital deaths was 3,687, with a mortality of 1.6%. The patient collection process is illustrated in Fig. 1.

### Characteristics of the study population

The results of the comparison of the characteristics between the survival and in-hospital death groups are presented in Table 1. Compared with the survival groups, the in-hospital death group was older and had a higher proportion of male patients (*p* < 0.001). This group also exhibited a higher percentage of high-risk mechanisms, such as traffic accidents and falls, as well as a higher incidence of TBI (*p* < 0.001). The EMR-ISS was significantly higher in the in-hospital death group at 41 (IQR 25, 66), compared with 9 (IQR 8, 18) in the survival group (*p* < 0.001). The rSIG value in the in-hospital death group was 10.84 (IQR 5.50, 19.84) lower than 24.03 (IQR 24.45, 28.21) in the survival group (*p* < 0.001).

**Table 1:** Characteristics of the study population

Variables	Survival group (*n* = 314,819)	In-hospital death group (*n* = 3,687)	*p*-value
Time from injury to ED arrival (min)	41 (30, 60)	39 (28, 60)	< 0.001
Age (years)	51 (35, 64)	68 (54, 77)	< 0.001
Male	193,603 (61.5)	2,535 (68.8)	< 0.001
Patients with TBI	52,141 (16.6)	2,236 (60.6)	< 0.001
Trauma mechanism			< 0.001
Traffic accident	118,924 (37.8)	1,833 (49.7)	
Fall down injury	117,431 (37.3)	1,657 (44.9)	
Blunt injury	54,843 (17.4)	128 (3.5)	
Penetrating injury	20,429 (6.5)	56 (1.5)	
Other^a^	3,192 (1.0)	13 (0.4)	
SBP (mmHg)	136 (120, 152)	130 (100, 160)	< 0.001
SBP < 90, n (%)	3,737 (1.2)	715 (19.4)	< 0.001
DBP (mmHg)	80 (70, 90)	80 (60, 95)	< 0.001
HR (beats/min)	83 (75, 94)	90 (75, 110)	< 0.001
HR > 100, n (%)	54,339 (17.3)	1,342 (36.4)	< 0.001
HR < 60, n (%)	6,836 (2.2)	250 (6.8)	< 0.001
RR (breath/min)	20 (18, 20)	20 (18, 22)	< 0.001
BT (°C)	36.5 (36.3,3 6.8)	36.2 (36.0, 36.6)	< 0.001
GCS	15 (15, 15)	8 (3,15)	< 0.001
GCS < 9, n (%)	3,254 (1.0)	1,904 (1.6)	< 0.001
EMR-ISS	9 (8, 18)	41 (25,66)	< 0.001
rSIG	24.03 (24.45, 28.21)	10.84 (5.50, 19.84)	< 0.001

Frequency (%) or median (interquartile range); *ED* Emergency departments, *TBI* Traumatic brain injury, *SBP* Systolic blood pressure, *DBP* Diastolic blood pressure, *HR* Heart rate, *RR* Respiratory rate, *BT* Body temperature, *EMR-ISS* Excess mortality ratio-adjusted injury severity score, *rSIG* Reverse shock index multiplied by Glasgow Coma Scale

^a^Other contains the low-frequency injury mechanisms, such as injury by machine

### Variables contributing to in-hospital mortality

The results of the logistic regression analysis to identify the factors contributing to in-hospital mortality are presented in Table 2. Multivariable regression analysis showed that the time from injury to ED arrival variable was not related to in-hospital mortality (*p* = 0.056), whereas other variables were associated with in-hospital mortality. rSIG was confirmed to have an independent association with in-hospital mortality (adjusted OR, 0.827 (95% CI 0.823–0.832)).

**Table 2:** Results of univariable and multivariable logistic regression analysis of in-hospital mortality

Variables	Univariable analysis		Multivariable analysis	
	OR (95% CI)	*p*-value	aOR (95% CI)	*p*-value
Time from injury to ED arrival	0.999 (0.998–1.000)	0.001	0.999 (0.998–1.000)	0.056
Male	1.378 (1.285–1.478)	< .001	1.117 (1.028–1.214)	0.009
Age (years)	1.041 (1.039–1.043)	< .001	1.055 (1.053–1.058)	< .001
Trauma mechanism				
Traffic accident	Reference			
Fall down injury	0.915 (0.856–0.979)	0.01	0.954 (0.878–1.035)	0.257
Blunt injury	0.151 (0.127–0.181)	< .001	0.365 (0.302–0.442)	< .001
Penetrating injury	0.178 (0.136–0.232)	< .001	0.347 (0.262–0.459)	< .001
Other^a^	0.264 (0.153–0.456)	< .001	0.546 (0.305–0.977)	0.042
EMR-ISS	1.072 (1.071–1.074)	< .001	1.044 (1.042–1.046)	< .001
rSIG	0.779 (0.775–0.782)	0.001	0.827 (0.823–0.832)	0.001

*OR* Odds ratio, *aOR* Adjusted odds ratio, *CI* Confidence interval, *ED* Emergency departments, *EMR-ISS* Excess mortality ratio-adjusted injury severity score, *rSIG* Reverse shock index multiplied by the Glasgow Coma Scale

^a^Other contains the low-frequency injury mechanisms, such as injury by machine

### Cutoff point for predicting in-hospital mortality using rSIG

**Fig. 2:**
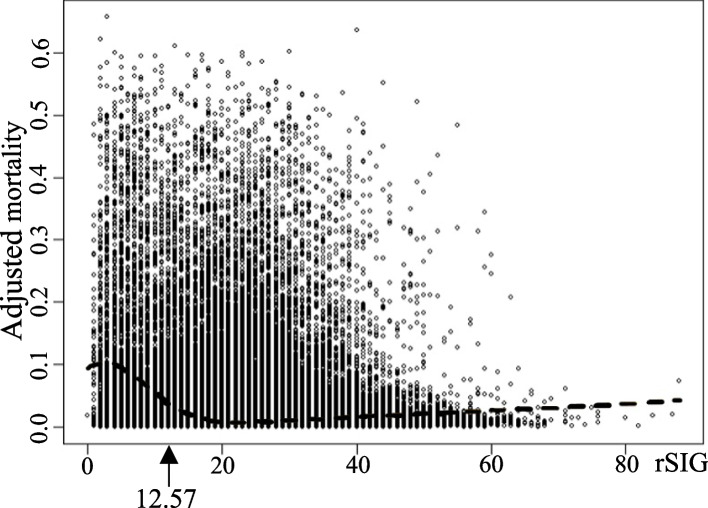
Plot depicting the correlation between rSIG and in-hospital mortality. Arrow: rSIG value at which the slope of the mortality showed the most significant increase. rSIG, reverse Shock Index Multiplied by the Glasgow Coma Scale

Figure 2 shows the correlation between rSIG and in-hospital mortality using the shape-restricted regression splines method. The in-hospital mortality began to increase as the rSIG value decreased to 18.86, with the most significant increase at 12.57. The increase in in-hospital mortality with decreasing rSIG values was observed from rSIG 20 and below. The cutoff value calculated using Youden's index was 16.5 (sensitivity, 0.663; specificity, 0.927).

Three cutoff points were applied to the entire patient group and the subgroup with rSIG 20 or less, where a correlation between the two variables was observed, and the over/under-triage rates were presented in Table 3. In all cases, the target over/under-triage rates set by the American College of Surgeons were not met. However, applying a cutoff point of 16.5 to the rSIG 20 or less group resulted in values that were the closest to the target classification criteria.

**Table 3:** Over-triage and under-triage rates for each rSIG cutoff

Target patient group	Statistics	Cutoff of rSIG		
		12.57	16.5	18.86
All patients	Over-triage rate (%)	1.96	7.37	16
	Under-triage rate (%)	44.40	33.50	27.2
rSIG is under 20	Over-triage rate (%)	8.50	33.46	72.65
	Under-triage rate (%)	27.38	11.89	3.56

*rSIG* Reverse shock index multiplied by Glasgow Coma Scale

**Fig. 3:**
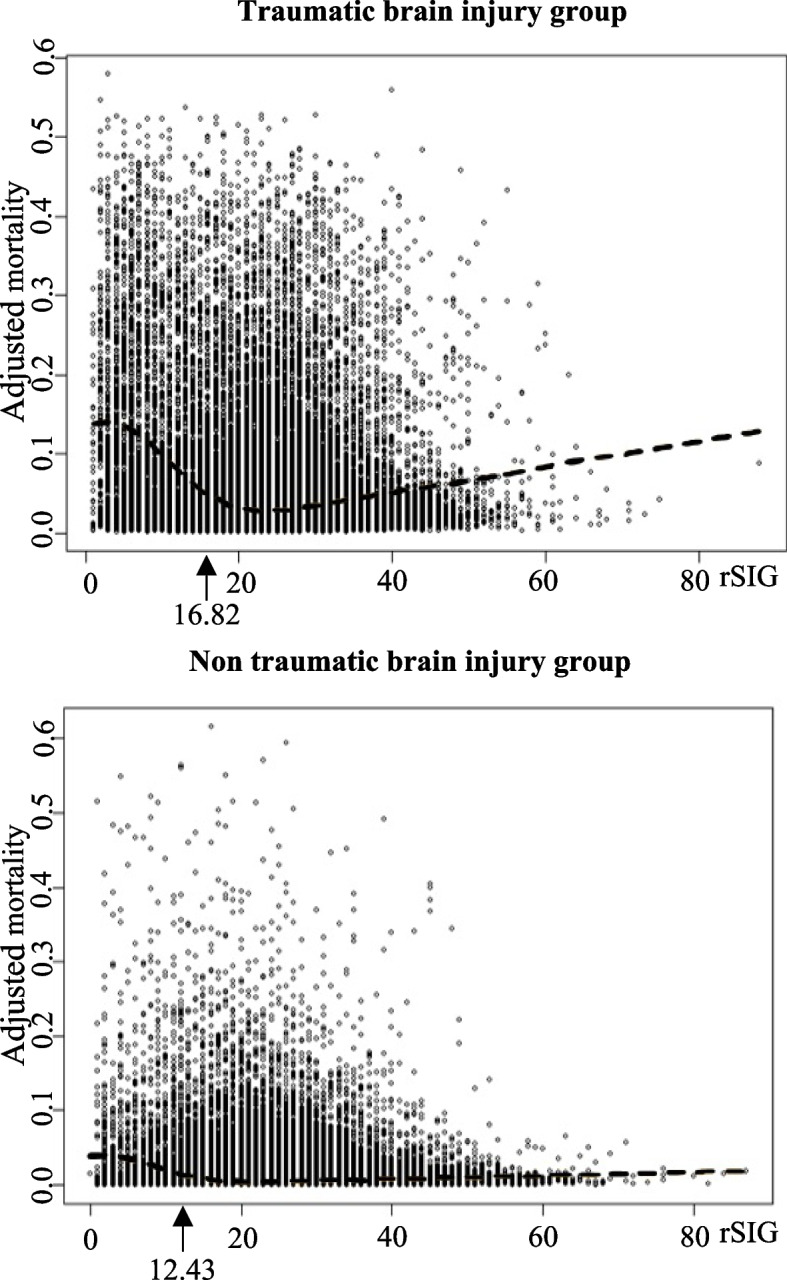
Plot of subgroup analysis for the correlation between rSIG and in-hospital mortality. Arrow: rSIG value at which the slope of the mortality showed the most significant increase. rSIG, reverse Shock Index Multiplied by the Glasgow Coma Scale

After categorizing the patients into two groups according to the presence or absence of TBI, Figure 3 illustrates the relationship between rSIG and in-hospital mortality in each group. The in-hospital mortality began to increase as the rSIG value decreased to 16.82 in TBI group and 18.64 in non-TBI group. Similar to the analysis results for the total patient group, the mortality started to increase from rSIG 20 or less, and in the TBI group, the increase in in-hospital mortality with decreasing rSIG was more pronounced. Additionally, different from the non-TBI group, the TBI group exhibited a unique trend in which in-hospital mortality increased as the rSIG value exceeded 25. The point of the most significant increase in in-hospital mortality was 16.82 in the TBI group and 12.43 in the non-TBI group. The cutoff points using Youden's index were 16.5 for the TBI group (sensitivity, 0.716; specificity, 0.892) and 17.5 for the non-TBI group (sensitivity, 0.619; specificity, 0.905).

### Subgroup analysis for sensitivity analysis

**Fig. 4:**
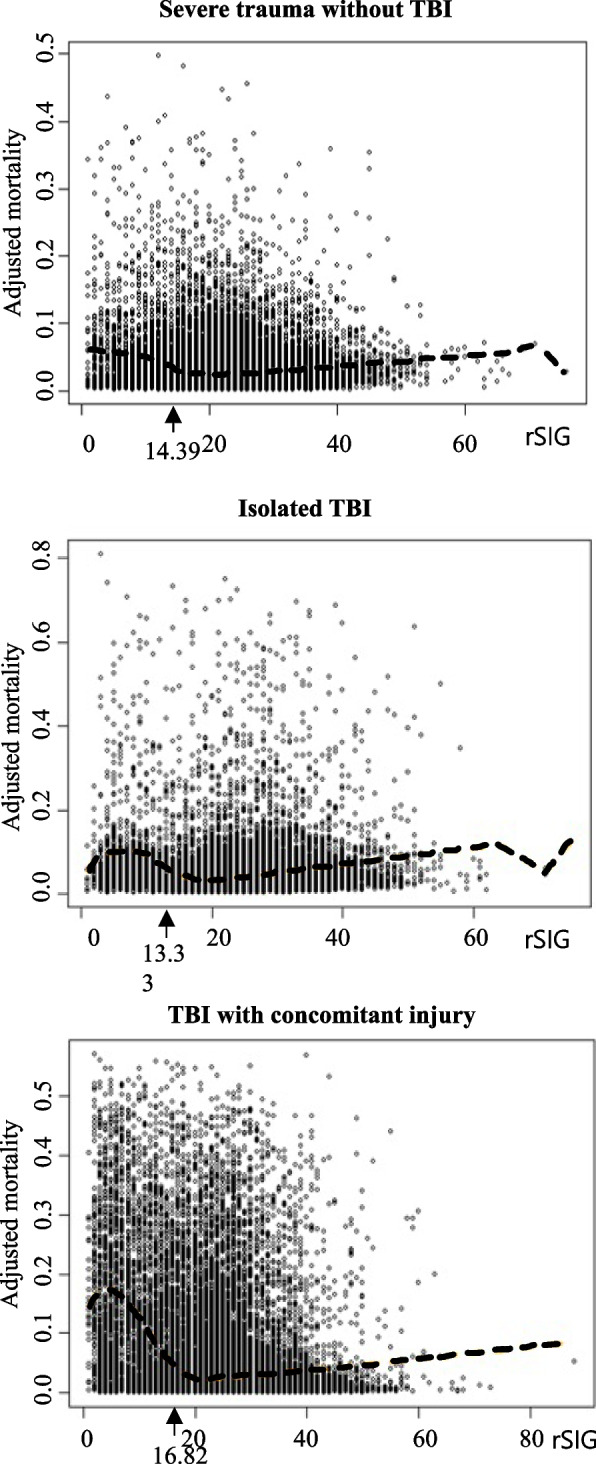
Plot of subgroup analysis for the correlation between rSIG and in-hospital mortality for sensitivity analysis. Arrow: rSIG value at which the slope of the mortality showed the most significant increase. TBI, traumatic brain injury; rSIG, reverse Shock Index Multiplied by the Glasgow Coma Scale

Owing to disparities in the correlation between in-hospital mortality and rSIG among the TBI and non-TBI groups, we conducted additional sensitivity analysis. We presented the shape-restricted regression splines method curves in Fig. 4. In the severe trauma without TBI group, the threshold at which in-hospital mortality began to rise as rSIG decreased was 22.43, whereas for the isolated TBI group, it was 22.58. For the TBI with other concomitant injuries group, the threshold was 24.73. The most significant changes in mortality rate occurred at 14.39, 13.33, and 16.82 for the severe trauma without TBI, isolated TBI, TBI with other concomitant injuries groups, respectively; the cutoff values derived using the Youden index were 16.5 (sensitivity, 0.698; specificity, 0.853), 15.50 (sensitivity, 0.654; specificity, 0.923), and 16.50 (sensitivity, 0.736; specificity, 0.889) for each respective group. In this subgroup analysis, akin to the findings of the previous analysis, a trend was observed wherein the in-hospital mortality rate tends to increase as rSIG decreases from around rSIG 20 or below. Furthermore, when comparing the cutoffs calculated using the Youden index for the entire patient population, a slightly lower value of 15.5 was noted in the isolated TBI group, whereas a consistent cutoff value of 16.5 was determined for the remaining groups.

### ROC curves and AUC values for each analysis

**Fig. 5:**
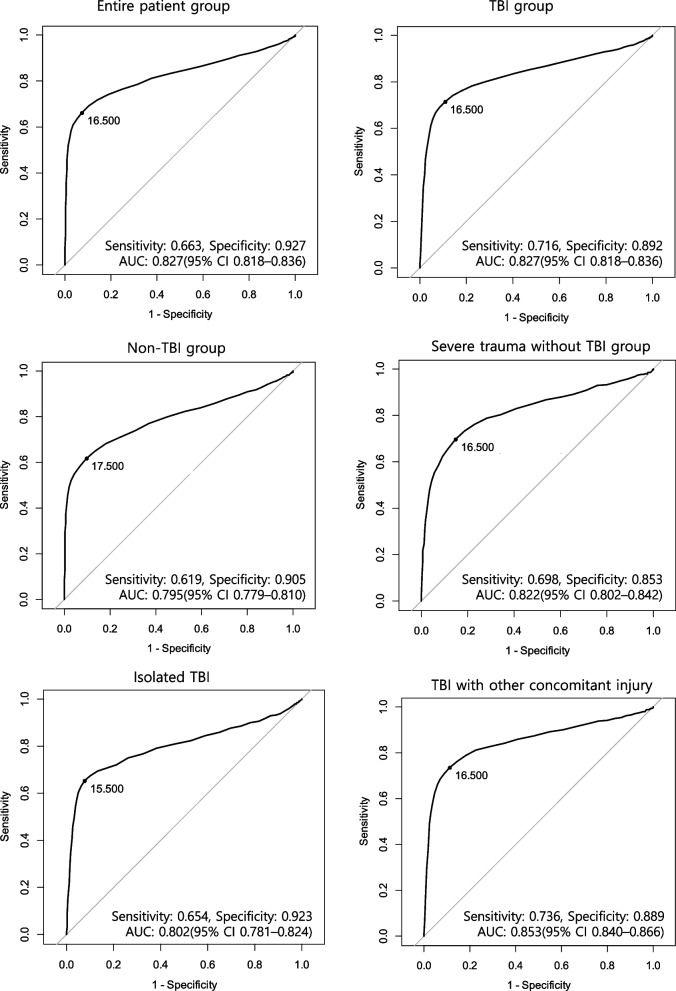
Receiver operating characteristic curves and area under the curve values for each analysis. TBI, traumatic brain injury; AUC, area under the curve; 95% CI, 95% confidence interval

ROC curves and AUC values for predicting in-hospital mortality based on rSIG for the entire patient group and all subgroups are presented in Fig. 5. In the entire patients group, the AUC value was 0.827 (95% CI 0.818–0.836), indicating good predictive power of rSIG for in-hospital mortality. For the non-TBI group, the AUC value was 0.795 (95% CI 0.779–0.810), suggesting fair predictive power. However, in all other analyses, AUC values above 0.8 were observed, indicating overall good predictive power of rSIG for in-hospital mortality. Among these, the TBI with other concomitant injuries group showed the highest AUC value of 0.853 (95% CI 0.840–0.866).

## Discussion

This study’s results demonstrated that overall in-hospital mortality began to increase progressively as rSIG values decreased below 18.86, reaching the most significant increase at 12.57. When focusing on patients with rSIG values of 20 or below, the cutoff point of 16.5 closely approximated the ideal classification tool conditions. Additionally, in the TBI group, when the rSIG was over 25, there was a unique tendency for the in-hospital mortality to increase with increasing rSIG. Unlike previous studies that approached cutoff point determination using a dichotomous method, our study graphically depicted the trend of in-hospital mortality based on changing rSIG values, offering a distinct advantage.

One of the most widely used trauma assessment tools is the national field triage guideline (FTG), which determines the need for transfer to a trauma center by integrating physiological indicators such as GCS and vital signs, components of rSIG, and major anatomical injuries identified by EMS paramedics or physicians and the mechanism of injury [18]. While FTG may allow for a more precise evaluation compared with rSIG, we focused on rSIG because the diagnosis of major anatomical injuries estimated in the prehospital phase, which is essential in FTG classification, is not accurate. According to studies by Kirves et al. in 2010 and Deeb et al. in 2022, there was a significant discrepancy between the diagnosis of major anatomical injuries evaluated by prehospital medical staff and the final diagnosis confirmed in the hospital [19, 20]. In both studies, the estimated rate of pelvic injuries in the prehospital phase was very low. Such discrepancy may be attributable to the fact that the ability to estimate major injuries depends on the experience and knowledge of the rescuer, leading to a weakness where classification results may vary depending on the rescuer's ability. rSIG is superior to FTG in this regard; however, unlike FTG's simple assessment of vital signs and GCS, rSIG may have the disadvantage of requiring rescuers to perform mathematical calculations in a hectic rescue environment. Recently, the use of electronic prehospital patient care reports has become widespread [21]; entering the measurement into such tools can reduce the time spent by rescuers on mathematical calculations, thereby overcoming this weakness. Additionally, rSIG has the advantage of continuously comparing evaluation values from the prehospital phase to the hospital phase and monitoring the patient's condition.

The proposed cutoff value of 16.5 in our study may be subject to debate. Generally, an SI of 1 or higher is the cutoff point for predicting poor prognosis in trauma, and when substituted with rSI, it is 1 or less [22]. Considering that the normal upper limit of the GCS is 15, an rSIG score of 16.5 implies that patients have an alert mental status and a stable hemodynamic status. However, a systematic review and meta-analysis by Carsetti et al. in 2023, which analyzed 670,728 patients with trauma, suggested a cutoff of 0.804 for predicting mortality, which translates to 1.24 when converted to rSI [23]. Applying this cutoff to patients with alert mental status, the rSIG cutoff would be approximately 18.66. Therefore, we determined that rSIG values exceeding 15 could also be used as a cutoff to predict poor prognosis.

Several previous studies have proposed cutoff values for rSIG to predict in-hospital mortality in adult patients with trauma. Lee et al. analyzed patients with trauma who were admitted to a level 1 trauma center and proposed an rSIG cutoff of 10.20, while Wan-Ting et al. analyzed patients with trauma with severe TBI (injury severity score 16 or higher) and proposed a cutoff of 14.0 [11, 13]. The cutoff presented in our study (16.5) was higher than those in previous studies. This discrepancy may arise from differences in the severity of the study populations. Our study included all adult patients with trauma, including minor trauma, whereas previous studies included only patients meeting criteria for field triage guideline or injury severity score 16 or higher, likely comprising a population with lower rSIG values. The rSIG values of the study populations in previous studies were lower, with Lee et al. reporting 17.82 (IQR 11.82—23.57) and Wan-Ting et al. reporting 19.49 ± 18.10, both lower than 24.03 (IQR 24.45—28.21) in our study. Similar to our study population, Chen et al. analyzed all adult patients transported through emergency medical services. In their study, the mean rSIG value for the study group was 23.80 ± 8.17, and they proposed a cutoff of 18. These values closely resemble those observed in our study [14]. Our study aimed to propose an rSIG cutoff point to identify patients with trauma with the potential risk of death at the scene. Therefore, the cutoff point of 16.5 analyzed in all patients with trauma rather than selectively in patients with severe trauma may be more appropriately applied in the field triage phase.

As rSIG decreases to 18.86 or below, the in-hospital mortality increases. This suggests that patients in this category may directly face a rise in mortality due to the deterioration in vital signs and consciousness. Therefore, patients in this group require close observation of consciousness and vital signs until trauma evaluation is completed. The point at which the slope of the in-hospital mortality increases most significantly is at rSIG 12.57, and the in-hospital mortality for patients with rSIG 12.57 or below reaches 25.97%. Consequently, these patients require more intensive evaluation and treatment upon arrival at the hospital.

Analysis of the subgroups divided by the presence or absence of TBI revealed a greater increase in the in-hospital mortality with decreasing rSIG in the TBI group when the rSIG was 20 or less. (Fig. 3) Impaired consciousness in patients with trauma without TBI is commonly secondarily induced by shock or hypoxemia. In this group, impaired consciousness recovered through corrective interventions. However, in the TBI group, both shock and GCS, which constitute the rSIG, directly influenced the mortality. As mentioned earlier, GCS is closely related to the prognosis of patients with TBI [24]. Additionally, patients with TBI experience impaired autoregulation of the brain owing to increased intracranial pressure, and shock accelerates the decrease in brain perfusion, leading to irreversible global brain hypoxia [25, 26]. Therefore, the correlation between rSIG and the in-hospital mortality is likely greater in the TBI group. In the TBI group, rSIG values tended to increase beyond 25, accompanied by an increase in the in-hospital mortality; this tendency was not observed in the non-TBI group. This may be due to Cushing’s reflex, specifically in patients with TBI. Cushing’s reflex is defined as a situation where SBP rises while HR and respiratory rates decrease rapidly with a rapid increase in intracranial pressure [27]. Patients with severe TBI, at risk of rapid increases in intracranial pressure, may exhibit extremely elevated rSIG owing to this reflex. Therefore, when interpreting rSIG values in patients with trauma, it is essential to consider not only the conventional approach that low rSIG values increase mortality but also abnormally high rSIG values indicating poor prognosis, especially in patients suspected of having TBI. Although attempts were made based on this study’s data to derive an appropriate cutoff point for patients with TBI with high rSIG, it was unsuccessful in achieving an appropriate cutoff that closely approximated the targeted under/over-triage rate. Therefore, we hope that future studies will propose suitable cutoff values.

This study had several limitations. First, as this study was based on data from a single country, the results cannot be generalized to other countries. Second, among the patients who met the inclusion criteria, a large number (approximately 20%) were excluded because of missing essential information. It is possible that the results might have changed if excluded patients were included. Third, as the analysis was conducted with limited precollected information, additional variables influencing trauma mortality, such as patient comorbidities and anticoagulant use, were excluded. Finally, although this study aimed to establish a cutoff for rSIG for use in field triage, the actual rSIG values used in this study were collected at the hospital stage. Therefore, there may be a potential mismatch between pre-hospital rSIG and those collected at the hospital.

## Conclusions

To predict in-hospital mortality in adult patients with trauma, we propose a cutoff rSIG of 16.5 or below. However, in patients with TBI and high rSIG values, higher rSIG values were also associated with in-hospital mortality. We hope suitable cutoffs will be proposed for this group in the future.

## Data Availability

The Korea Disease Control and Prevention Agency (KDCA) prohibits the disclosure of study data collected through the EDIIS without permission. The data were licensed for the current study. Therefore, the data are not publicly available. However, these data are available upon reasonable request and with permission from the Korea Disease Control and Prevention Agency for academic purposes.

## Abbreviations

PTDR: Preventable trauma death rate
TBI: Traumatic brain injury
SI: Shock index
SBP: Systolic blood pressure
HR: Heart rate
rSI: Reverse Shock Index
GCS: Glasgow Coma Scale
95% CI: 95% Confidence intervals
IQR: Interquartile range
ROC: Receiver operating characteristic
AUC: Area under the curve
OR: Odds ratio
rSIG: Reverse shock index multiplied by GCS
EDIIS: Emergency Department-based Injury In-depth Surveillance
FTG: Field triage guideline
